# Molecular and Functional Changes to Postsynaptic Cholinergic Signaling in the Vestibular Sensory Organs of Aging C57BL/6 Mice

**DOI:** 10.1093/gerona/glad067

**Published:** 2023-02-25

**Authors:** Lauren A Poppi, Mark J Bigland, Ethan T Cresswell, Hessam Tabatabaee, David Lorincz, Hannah R Drury, Robert J Callister, Joseph C Holt, Rebecca Lim, Alan M Brichta, Doug W Smith

**Affiliations:** Neurobiology of Aging and Dementia and Vestibular Neurobiology Laboratories, School of Biomedical Sciences and Pharmacy, The University of Newcastle, Newcastle, New South Wales, Australia; Hunter Medical Research Institute, Newcastle, New South Wales, Australia; Neurobiology of Aging and Dementia and Vestibular Neurobiology Laboratories, School of Biomedical Sciences and Pharmacy, The University of Newcastle, Newcastle, New South Wales, Australia; Hunter Medical Research Institute, Newcastle, New South Wales, Australia; Neurobiology of Aging and Dementia and Vestibular Neurobiology Laboratories, School of Biomedical Sciences and Pharmacy, The University of Newcastle, Newcastle, New South Wales, Australia; Hunter Medical Research Institute, Newcastle, New South Wales, Australia; Neurobiology of Aging and Dementia and Vestibular Neurobiology Laboratories, School of Biomedical Sciences and Pharmacy, The University of Newcastle, Newcastle, New South Wales, Australia; Hunter Medical Research Institute, Newcastle, New South Wales, Australia; Neurobiology of Aging and Dementia and Vestibular Neurobiology Laboratories, School of Biomedical Sciences and Pharmacy, The University of Newcastle, Newcastle, New South Wales, Australia; Hunter Medical Research Institute, Newcastle, New South Wales, Australia; Neurobiology of Aging and Dementia and Vestibular Neurobiology Laboratories, School of Biomedical Sciences and Pharmacy, The University of Newcastle, Newcastle, New South Wales, Australia; Hunter Medical Research Institute, Newcastle, New South Wales, Australia; Neurobiology of Aging and Dementia and Vestibular Neurobiology Laboratories, School of Biomedical Sciences and Pharmacy, The University of Newcastle, Newcastle, New South Wales, Australia; Hunter Medical Research Institute, Newcastle, New South Wales, Australia; Department of Otolaryngology, University of Rochester Medical Center, Rochester, New York, USA; Neurobiology of Aging and Dementia and Vestibular Neurobiology Laboratories, School of Biomedical Sciences and Pharmacy, The University of Newcastle, Newcastle, New South Wales, Australia; Hunter Medical Research Institute, Newcastle, New South Wales, Australia; Neurobiology of Aging and Dementia and Vestibular Neurobiology Laboratories, School of Biomedical Sciences and Pharmacy, The University of Newcastle, Newcastle, New South Wales, Australia; Hunter Medical Research Institute, Newcastle, New South Wales, Australia; Neurobiology of Aging and Dementia and Vestibular Neurobiology Laboratories, School of Biomedical Sciences and Pharmacy, The University of Newcastle, Newcastle, New South Wales, Australia; Hunter Medical Research Institute, Newcastle, New South Wales, Australia

**Keywords:** Cholinergic, Efferent, Hair cell, Nicotinic, Vestibular

## Abstract

Cholinergic circuits in the central nervous system are vulnerable to age-related functional decline, but it is not known if aging impacts cholinergic signaling in the vestibular sensory organs, which are critically important to balance maintenance and visual gaze stability. We have previously shown cholinergic neurotransmission between vestibular efferent terminals and type II mechanosensory hair cells requires the alpha9 (*Chrna9*) nicotinic receptor subunit. Homozygous knockout of the alpha9 subunit causes vestibulo-ocular reflex adaptation deficits that mirror those observed in aged mice. This prompted examination of cholinergic signaling in the vestibular sensory organs of aged mice. We confirmed older (>24 months) mice had impaired performance in a balance beam task compared to young (3–4 months) adult mice. While there was no qualitative loss of cholinergic axon varicosities in the crista ampullaris of old mice, qPCR analysis revealed reduced expression of nicotinic receptor subunit genes *Chrna1*, *Chrna9*, and *Chrna10* in the cristae of old relative to young mice. Functionally, single-cell patch clamp recordings taken from type II vestibular hair cells exposed to acetylcholine show reduced conductance through alpha9/10 subunit-containing nicotinic receptors in older mice, despite preserved passive membrane properties and voltage-activated conductances. These findings suggest that cholinergic signaling in the peripheral vestibular sensory organs is vulnerable to aging processes, manifesting in dynamic molecular and functional age-related changes. Given the importance of these organs to our everyday activities, and the dramatic increase in fall incidence in the older, further investigation into the mechanisms of altered peripheral vestibular function in older humans is warranted.

## Graphical Abstract

**Graphical Abstract:**
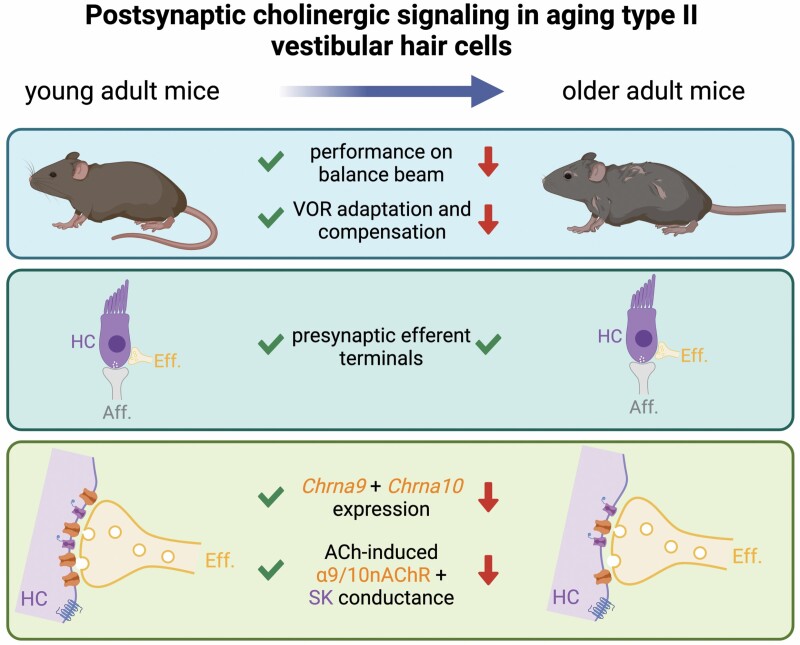

Sensory systems functionally decline with age, and each sensory system is believed to have a unique neuropathological trajectory. Age-related vestibular dysfunction is a major health problem associated with life-threatening injuries and loss of independence in older adults, but the range of mechanisms underlying these changes is not well understood (1). In humans, the loss of mechanosensory hair cells (HCs) (2,3), primary afferent somata (4), vestibular nerve fibers (5), and central vestibular neurons (6) have all been linked to aging. However, animal models of aging suggest that peripheral deficits arise principally from altered mechanotransduction or synaptic transmission as opposed to HC loss (7). On the other hand, genetic models thought to recapitulate aspects of aging, that is, *Bdnf* knockout mice, show loss of vestibular ganglion neurons and HCs (8). Although expression of *Bdnf* and its receptors appear to be important for the survival of sensory afferents (9), it is not known if *Bdnf* and/or Trk receptor expression changes drive vestibular functional decline in normal aging.

A clinical measure of peripheral vestibular function, the vestibulo-ocular reflex (VOR), is remarkably intact in humans up to 89 years of age (10). However, after normalizing for VOR gain, the amplitude of compensatory catch-up saccades correlates with increasing age (11). Similarly, aged mice show normal VOR at baseline, but VOR adaptation is significantly impaired in older mice compared to young adult controls (12).

The efferent vestibular system (EVS) originates in the brainstem and projects to the peripheral vestibular sensory organs, contacting both HCs and primary afferents (13,14). The precise role of the primarily cholinergic EVS is still not well understood, but it is a highly conserved circuit and is thought to be functionally important (15,16). Acetylcholine (ACh) has a robust effect on the activity of vestibular HCs (17–19) and primary afferent neurons (20). ACh acts on vestibular type II HCs via nicotinic acetylcholine receptors containing α9 and α10 subunits (18,19). The α9 subunit (encoded by *Chrna9*) commonly associates with the α10 subunit (encoded by *Chrna10*) to form functional heterodimeric receptors (21). Like aged mice (12), homozygous *Chrna9* knockout mice showed deficits in VOR adaptation (22). This prompted us to examine cholinergic efferent synapses in the vestibular sensory organs of aging mice.

Expression of cholinergic receptors is known to be important for the development of sensory networks (23,24). In addition, cholinergic circuit changes are related to both aging and neurodegeneration (25). While it has been shown in older adult gerbils that the number of cholinergic vestibular efferent neurons in the brain does not decrease with age (26), peripheral efferent synapses have not been examined. Based on observations of VOR adaptation in both homozygous *Chrna9* knockout and aged mice, we hypothesized that aging impacts cholinergic signaling between efferent neurons and type II vestibular HCs. We present an initial anatomical, molecular, and electrophysiological assessment of the impacts of aging on peripheral EVS function in C57BL/6 mice.

## Method and Materials

### Animal Ethics

All procedures were conducted in accordance with the Australian Code for the Care and Use of Animals for Scientific Purposes, 8th Edition, 2013, the NSW Animal Research Act and Regulations, and were approved by the University of Newcastle Animal Care and Ethics Committee, protocol numbers A-2013-325 and A-2011-117.

### Aging Mouse Colony

Male C57BL/6 mice were group housed with controlled temperature and humidity, a standard 12-hour light/dark cycle, and ad libitum access to normal chow and water. Unless otherwise stated, 3 age groups were used for this study: juvenile (21–28 days; mean age 24.8 days); young adult (3–6 months; mean age 4.5 months); and aged mice (>24 months of age; mean age 27.9 months).

### Balance Beam Task

**Figure 1.**
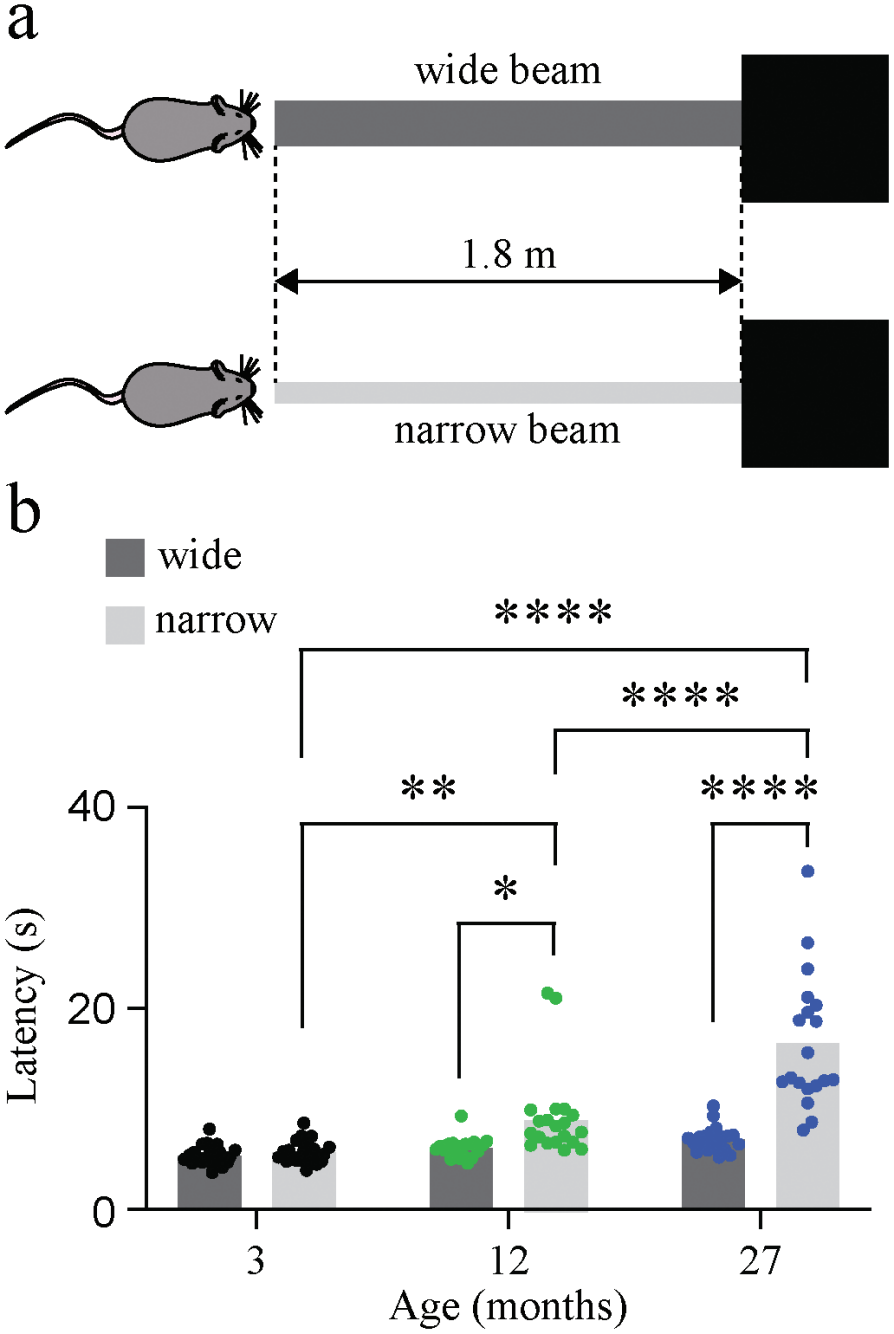
Aging C57BL/6 mice show impaired performance on a balance beam task. Schematic of balance beam task - each mouse made 3 attempts at crossing a wide (30 mm) beam, and a narrow (12 mm) beam to enter a dark box at the end of the beam (A). Beam cross latency of ~3-month young adult (*n* = 24), ~12 month middle-aged adult (*n* = 20) and ~27-month aged adult (*n* = 21) C57BL/6 male mice across a wide beam (left bars) and a narrow beam (right bars) (B). Two-way ANOVA with Šídák’s multiple comparisons test. ANOVA = analysis of variance.

Male C57BL/6 mice from 3 age groups (young adult: 3 months, adult: 12 months, and aged: >24 months) were filmed at 30 fps while crossing a 1.8-m-long balance beam that was either 30 mm (wide) or 12 mm (narrow) in width, with a safety dark box at the end of the beam (Figure 1A). The beam was elevated 60 cm above the ground (27). Each mouse was given 3 trials at crossing each beam width. Latency was measured from the time at which the mouse began to advance across the beam to the time the mouse reached the dark box at the end of the beam. Values were presented as the average latency for each animal. Graphing and statistical testing were done in GraphPad Prism (v. 9.4.0, GraphPad Software, San Diego, CA).

### Immunofluorescence

Male C57BL/6 mice were anesthetized with intraperitoneal ketamine (100 mg/kg) and medetomidine (1 mg/kg), and transcardially perfused with ice-cold phosphate-buffered saline (PBS) prior to rapid temporal bone dissection. Bony labyrinths were drop-fixed in 4% paraformaldehyde in 0.1 M PBS for 2 hours at room temperature. Samples were rinsed in PBS, and then stored in PBS + 0.01% sodium azide at 4 °C for up to 1 week until dissection. Dissected cristae were embedded in 4% agarose (Sigma, Darmstadt, Germany) and sectioned at 40 µm (Leica CM1950, Leica, Wetzlar, Germany). Sections were blocked in 10% normal donkey serum (Sigma) for 1 hour prior to incubation in primary antibodies at room temperature for 2 nights (goat anti-ChAT, 1:100, Millipore, Burlington, MA, USA; rabbit anti-myosin VIIa, 1:200, Proteus, Proteus BioSciences, Ramona, CA, USA; and chicken anti-neurofilament H, 1:500, Merck, Darmstadt, Germany). Sections were washed in PBS and incubated in secondary antibodies at room temperature for 2 hours (donkey anti-goat Alexa-488 conjugate, 1:50, Abcam, Cambridge, UK; donkey anti-rabbit Alexa-594, 1:50, Jackson IR, West Grove, PA, USA; donkey anti-goat Alexa-594, 1:50, Abcam; donkey anti-rabbit Alexa-405, 1:200, Abcam; and donkey anti-chicken Alexa-488, 1:200, Jackson IR). Sections were mounted onto slides in SlowFade Gold mounting media (ThermoFisher, Waltham, MA) and imaged on a Nikon Eclipse C1 (Nikon, Tokyo, Japan) or Zeiss LSM900 confocal microscope using 60× and 100× oil immersion objective lenses. Images were postprocessed in Image J.

### Gene Expression

Male C57BL/6 mice were lethally anesthetized with Lethabarb (1 mg/kg; Virbac, Carros, France) and transcardially perfused with ice-cold DEPC-treated PBS. Cristae were dissected in ice-cold PBS as described previously (28), snap frozen in RNA later, and stored at −80°C until processing. Cristae were thawed and homogenized in RNA lysis buffer. RNA was extracted using a single-cell RNA purification kit (Norgen, Norgen Biotek, Thorold, Canada). RNA was then quantified using the NanoDrop 1000, DNAse 1 treated (Invitrogen), and reverse transcribed using a Sensifast cDNA synthesis kit (Bioline, Memphis, TN, USA). qPCR primers were designed using Primer-BLAST and tested to ensure single-peak melt curves and primer efficiencies of at least 80%. See Supplementary Table 1 for descriptions and aliases of genes assayed and primer sequences. Primer efficiencies were determined using 10-fold dilution series with 4 different cDNA concentrations and the equation: 10^(−1/slope of dilution curve) −1. qPCR reactions were carried out on an ABI 7500, using 12 µL reaction volumes and the SensiFAST SYBR Lo-ROX Kit (Bioline, Applied Biosystems, ThermoFisher, Waltham, MA, USA). Average cycle threshold (Ct) values of each gene were calculated using 7500 SDS software v2.0.6 and are shown in Supplementary Table 1. For the reference Ct, the geometric mean of the Cts for *Actb*, *Gapdh*, and 28srRNA was used (29). Relative expression for each gene was determined by the comparative Ct method (30) and for graphing, data were normalized to expression in the young adult cohort.

### Electrophysiology

Male C57BL/6 mice were overdosed with intraperitoneal ketamine (100 mg/kg). Inner ears were dissected and vestibular neuroepithelium prepared for recordings as described previously (28) (Figure 5A). Borosilicate micropipettes (3–5 MΩ; King Precision Glass, Inc., Claremont, CA) were filled with KCl.gluconate internal solution containing (in mM) 42 KCl, 98 K-gluconate, 4 HEPES, 0.5 EGTA, 1 MgCl_2_, and 5 Na.ATP. Recordings were done in oxygenated L-15 medium (Gibco, Billings, MA, USA) at room temperature. Type II HCs were identified by their cylindrical top-down morphology and signature current responses to a voltage ladder ranging from −124 to +16 mV (Figure 4A). The voltage ladder protocol (Figure 4A) provided the following information: current–voltage (*I*/*V*) relationships, activation curves, peak currents at +16 mV, tail currents at −34 mV, and evidence of possible HCN (*I*_H_), and inward rectifier (*I*_KIR_) current, specifically from the −124 mV hyperpolarizing step. *I*_H_ current amplitude was measured within ~60 seconds of membrane breakthrough. Using alternating steps of +6 mV and −14 mV, the series resistance (*R*_s_), membrane resistance (*R*_m_), and membrane capacitance (*C*_m_) of each cell were measured at the beginning and end of each recording. If *R*_s_ values were >20 MΩ or changed more than 20% during the recording session, data were excluded from analysis. Acetylcholine (ACh; Sigma) was dissolved in L-15 medium (300 µM) and applied exogenously via a picospritzer for 100 milliseconds (18) (Figure 5A). ACh-activated currents were recorded at various potentials (*V*_m_ = −44 mV, −64 mV, and −94 mV). Antagonists (strychnine; 1 µM; Sigma-Aldrich; and apamin; 100 nM; Alomone, Jerusalem, Israel) were bath applied. Voltages have been corrected for liquid junction potential (4 mV for KCl.gluconate). Three repetitions of each response were averaged to produce traces shown. Data were collected with Multiclamp 700B and Axopatch 1D amplifiers (Molecular Devices, San Jose, CA, USA). Signals were sampled at 20 kHz, filtered at 10 kHz, and digitized using Instrutech ITC16/USB16 A-D boards (InstruTech, Lambrecht, Rheinland-Pfalz, Germany). Acquisition and analysis were done in AxographX (Axograph, Sydney, Australia). For each ACh response, the peak amplitude, rise time (10%–90% of peak), width (at 50% peak), decay (100%–50% of peak), and the absolute area under the curve (charge; pA/s) were measured. At holding potentials of −64 and −44 mV, where there are both negative- and positive-going currents, the area under the curve was measured as the sum of the absolute area, that is, |negative current area| + |positive current area| (see inset, Figure 5D). Graphing and statistical analyses were done in GraphPad Prism (GraphPad, San Diego, CA, USA). Some data from the juvenile group have been published previously as a part of another study (18).

**Figure 2.**
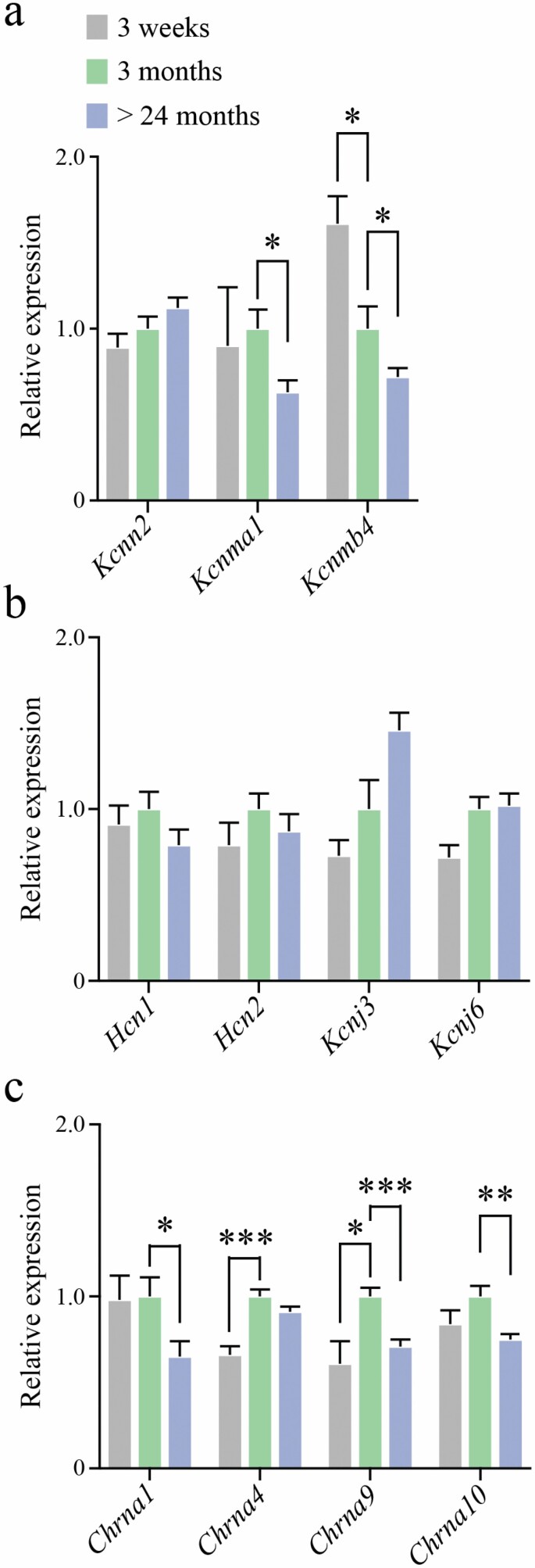
Gene expression changes in the cristae of aging C57BL/6 male mice. Relative gene expression in juvenile (P21, *n* = 8), young adult (3M, *n* = 8), and aged (27M, *n* = 8) vestibular neuroepithelium as determined by RT-qPCR. Genes were grouped into 3 functional categories: Ca^2+^-activated potassium channels (A), voltage-activated channels (B), and nicotinic acetylcholine receptor subunits (C). *t* Tests with multiple comparison correction*. Note*: Comparisons between aged and juvenile mice were not performed.

**Figure 3.**
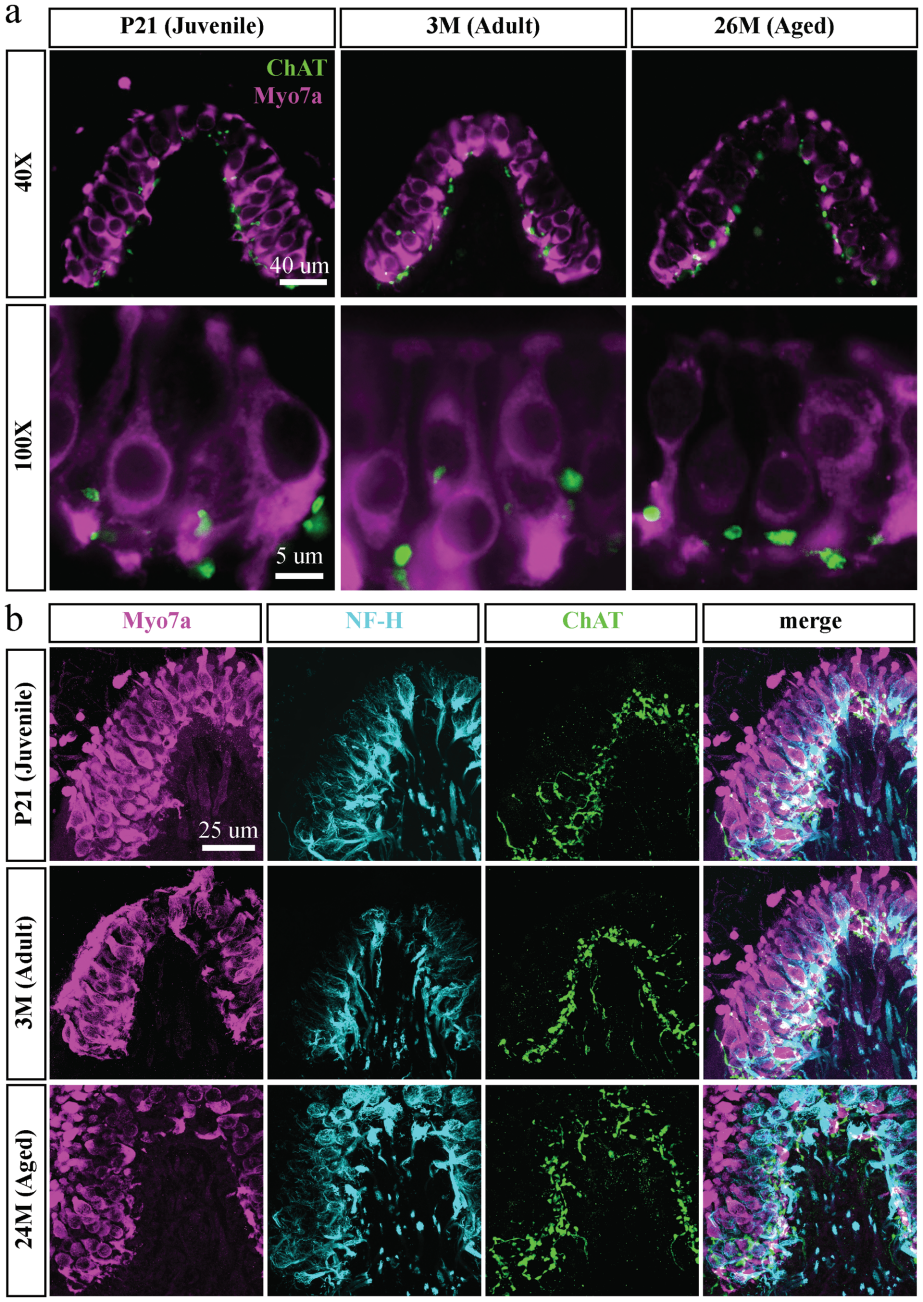
Aging C57BL/6 mice do not show marked loss of efferent varicosities in the crista ampullaris. Representative confocal z-stack images of cholinergic (ChAT) axon terminals in the cristae ampullares of juvenile (P21), young adult (3M), and aged (24M) C57BL/6 male mice with respect to vestibular sensory hair cells (Myo7a) (A). Representative images of crista ampullaris in juvenile (P21), young adult (3M), and aged (24M) C57BL/6 mice labelling hair cells (Myo7a, first column), afferent endings (NF-H, second column), and efferent varicosities (ChAT, third column), with composite images (fourth column) (B).

## Results

### Behavioral Assessment—Balance Beam Task

The latency of 3-month-old (3M; *n* = 24), 12-month-old (12M; *n* = 20), and 27-month-old (27M; *n* = 21) mice crossing a wide and a narrow balance beam across 3 trials was recorded (see schematic, Figure 1A). There was no significant effect of age on the ability to navigate the wide beam (Figure 1B; 3M vs 12M *p* = .9998, 12M vs 27M *p* = .7769), but aging did have a significant effect on latency to cross the narrow beam (Figure 1B; 3M vs 12M *p* = .005; 3M vs 27M *p* < .0001; 12M vs 27M *p* < .0001). Although 3M mice navigated both beams with similar latency (*p* > .05), 12M and 27M mice showed deficits in navigating the narrow relative to the wide beam (*p* = .038, and *p* < .0001, respectively). Data were statistically compared using 2-way analysis of variance (ANOVA) with Šídák’s multiple comparisons correction.

### Molecular Assessment—Gene Expression

Assayed genes (Figure 2) were grouped into 3 functional categories for ease of discussion: Ca^2+^-activated K^+^ channels (*Kcnn2*, *Kcnma1*, and *Kcnmb4*; Figure 2A), voltage gated-like (VGL) channels (*Hcn1*, *Hcn2*, *Kcnj3*, and *Kcnj6*; Figure 2B), and cholinergic receptor subunits (*Chrna1*, *Chrna4*, *Chrna9*, and *Chrna10*; Figure 2C). In the Ca^2+^-activated K^+^ channel group, aged mice (*n* = 8) showed a significant decrease in expression of both *Kcnma1* (*p* = .011) and *Kcnmb4* (*p* = .034), but no significant change in expression of *Kcnn2* (*p* > .05) compared to young adult mice (*n* = 8). In addition, there was a decrease in *Kcnmb4* expression in the maturation period from juvenile (*n* = 8) to young adult mice (*p* = .011). In the VGL channel group, there were no significant changes (*p* > .05) in *Hcn1*, *Hcn2*, *Kcnj3*, or *Kcnj6*. In the cholinergic receptor subunit group, there was a significant reduction in the expression of *Chrna1* (*p* = .021), *Chrna9* (*p* < .001), and *Chrna10* (*p* = .004), but no change in *Chrna4* with aging. There was a significant increase in *Chrna4* expression (*p* < .001), and a significant increase in *Chrna9* expression (*p* = .041) from juvenile to young adult. Average Ct values are shown in Supplementary Table 1.

### Neuroanatomical Assessment—Efferent Terminal Immunofluorescence

We observed no gross qualitative anatomical differences in ChAT-positive varicosities of juvenile (*n* = 4), young adult (*n* = 4), and aged (*n* = 4) C57BL/6 cristae (Figure 3). ChAT-positive terminals were present in the cristae of mice at all 3 ages, with similar distributions and sizes.

### Electrophysiological Assessment—Voltage-Activated Currents

**Figure 4.**
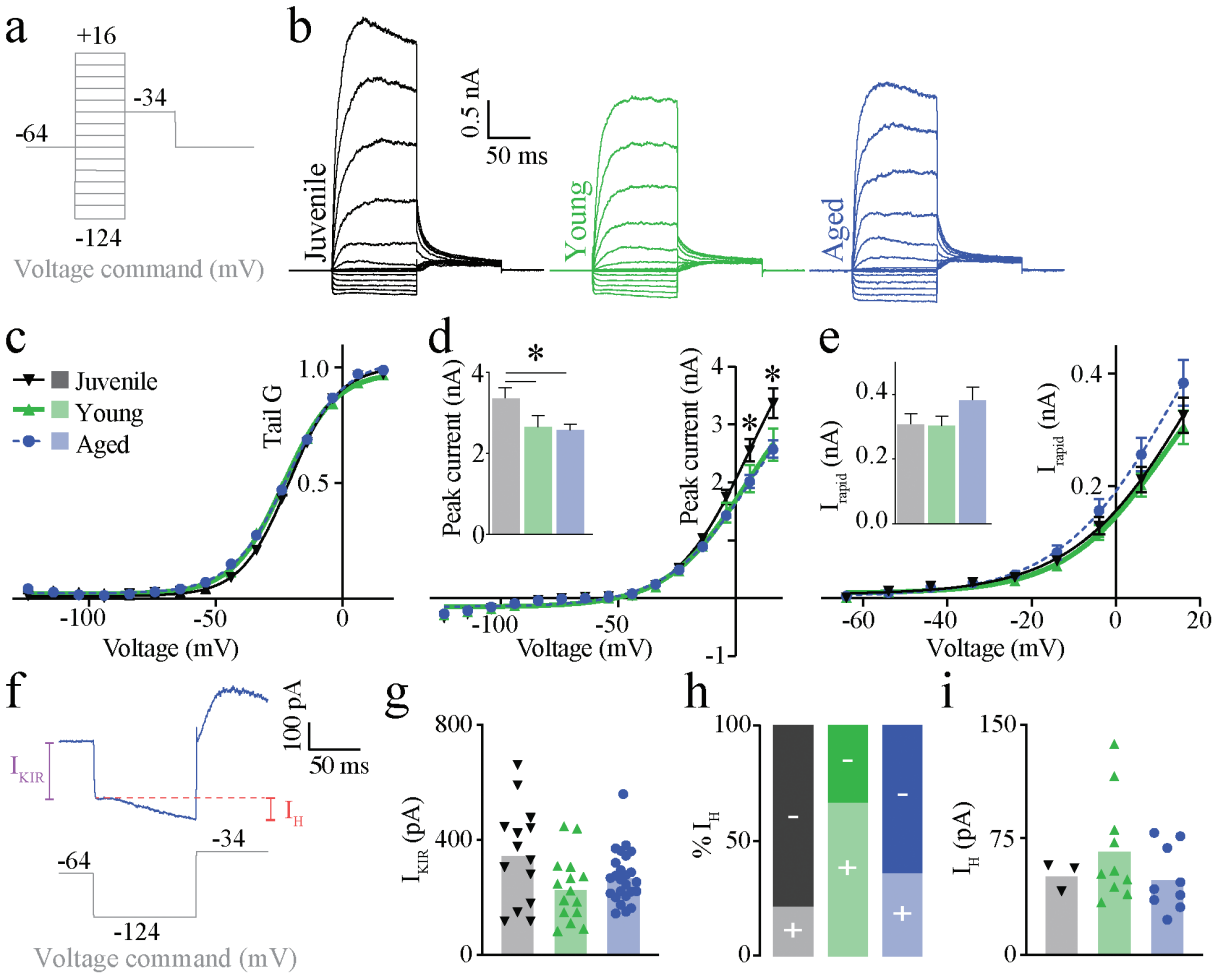
Voltage-activated currents of type II hair cells are not significantly impacted by aging. Voltage command “ladder” protocol used to measure voltage-activated currents. Voltages have been corrected for liquid junction potentials (A). Representative type II hair cell responses to voltage ladder. Juvenile (left trace), young adult (middle trace), and aged (right trace) (B). Normalized tail conductance curves for juvenile, young adult, and aged mice (*p* > .05 nonlinear regression) (C). Peak voltage-activated currents relative to membrane voltage (command). Inset shows peak current @ *V*_m_ = +16 mV (*p* < .05 multiple *t*-tests) (D). *I*–*V* curves for rapid voltage-activated current (*I*_rapid_) measured @ 1 ms current principally mediated by big conductance Ca^2+^-activated K^+^ (BK) channels. Inset shows *I*_rapid_ amplitude measured at *V*_m_ = +16 mV (*p* > .05; 2-way ANOVA) (E). Representative trace showing an aged type II HC response to the −124 mV hyperpolarizing step, where competing conductances *I*_KIR_ and *I*_H_ were measured (F). *I*_KIR_ amplitudes (*p* > .05; two-way ANOVA) (G). Relative proportion of type II hair cells recorded with (+) and without (−) measurable *I*_H_ current (H). Amplitudes of *I*_H_ currents in type II hair cells measured @ 100 ms (*p* > .05; 2-way ANOVA) (I). ANOVA = analysis of variance.

The mean *R*_m_ (529.7 MΩ) and mean *C*_m_ (5.8 pF) of aged type II HCs were comparable to values reported previously in type II HCs from young wild-type mice (18). Voltage-activated currents were unaffected by aging, with similar peak voltage-activated currents between aged and young adult type II HCs (Figure 4B). Juvenile type II HCs had larger peak current amplitudes on average (3.7 ± 0.25 nA, *n* = 14; Figure 4B and D) compared to type II HCs from both young adult (2.65 ± 0.28, *n* = 15, *p* < .05) and aged animals (2.58 ± 0.15, *n* = 25, *p* < .05). Tail currents measured at −34 mV (Figure 4C) were similar across aged (*n* = 25), young adult (*n* = 15), and juvenile (*n* = 14) type II HCs (*p* > .05; nonlinear regression). Due to decreased *Kcnma1* expression in aged cristae (Figure 2A), we measured rapidly activating K^+^ current at 1 millisecond after the onset of the depolarizing step which would capture mostly BK channels (31). Rapidly activating *I*/*V* plots are shown in Figure 4E. There was no significant difference in rapidly activating (likely BK) currents (*p* > .05; nonlinear regression). We also examined *I*_H_ and *I*_KIR_ currents in type II HCs (Figure 4F–I). *I*_KIR_ was measured using the −124 mV voltage step (Figure 4F). There was no significant change in *I*_KIR_ amplitude across the 2 age groups (Figure 4G). *I*_H_ was measured using the −124 mV voltage step of 100 milliseconds duration (Figure 4F). A total of 3/14 juvenile (21%) type II HCs, 10/15 (67%) young adult type II HCs, and 9/25 (36%) aged type II HCs exhibited a measurable *I*_H_ current at 100 milliseconds (Figure 4H). There were no significant differences in *I*_H_ amplitude across the 3 age groups (Figure 4I).

### Electrophysiological Assessment—Acetylcholine-Evoked Currents

**Figure 5.**
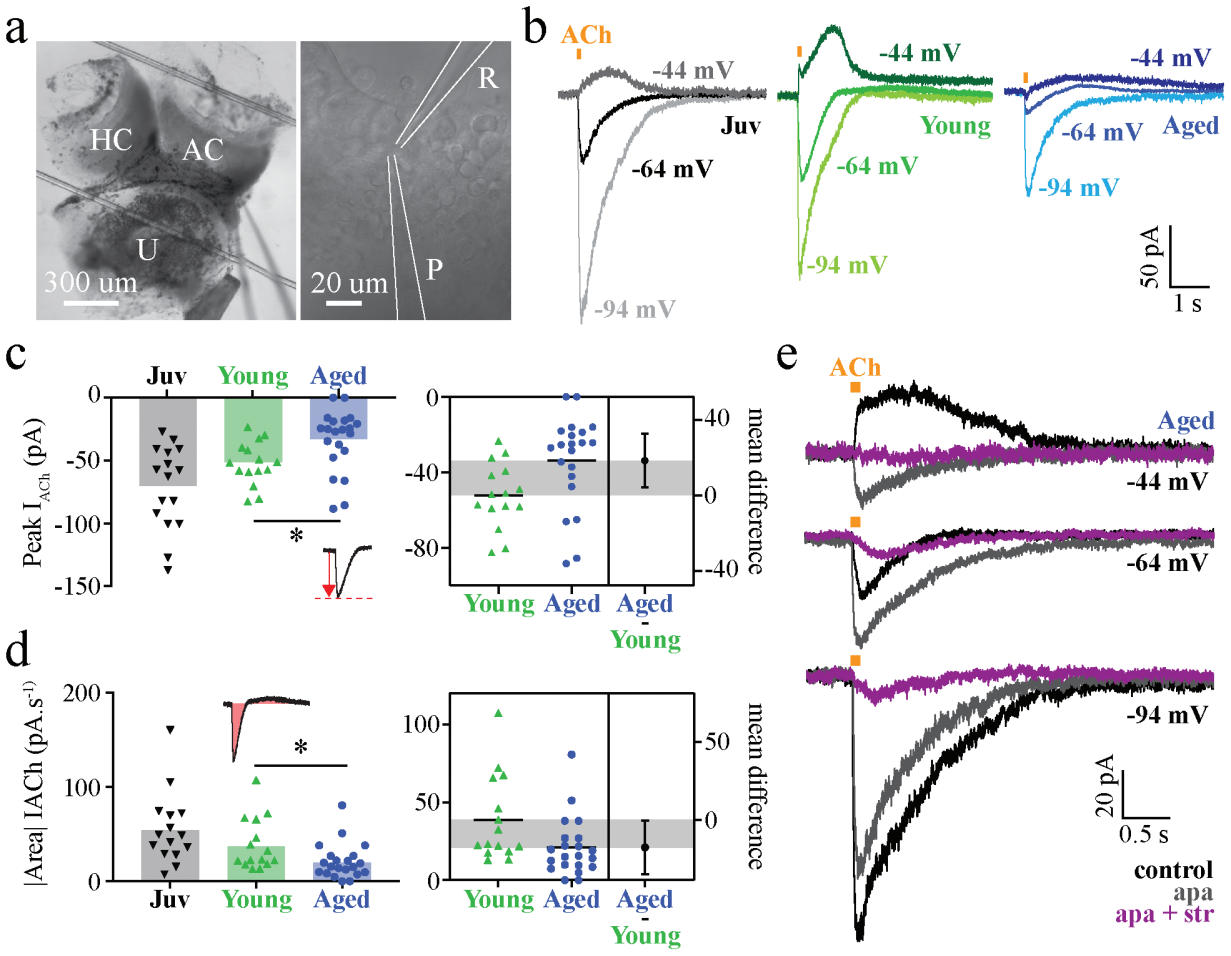
Type II vestibular hair cells in aged mice show reduced ACh-evoked conductances via α9/10 nicotinic ACh receptors. Vestibular neuroepithelium preparation for recordings (left). DIC image (right) showing hair cells of the anterior crista with recording (R) and puff (P) electrodes in place (A). Representative traces showing whole-cell voltage clamp recordings in response to locally applied ACh (300 µM; 100 ms) in external media and respective current–voltage relationships at −44 mV, −64 mV, and −94 mV. Each trace shown is the average of 3 consecutive repetitions, with 60 s start-to-start intervals (B). Group data (left) for peak ACh-induced current amplitudes (inset schematic) at *V*_m_ = −64 mV in juvenile (*n* = 16), young adult (*n* = 15), and aged (*n* = 21) type II hair cells (Brown–Forsythe and Welch ANOVA, age group effect, *p* = .0005; multiple *t*-tests, young vs. aged, *p* = .0121). Estimation plot (right) for young vs. aged adult type II hair cell peak amplitudes (C). Group data (left) for total current area (inset schematic) of type II hair cell responses at *V*_m_ = −64 mV in juvenile (*n* = 15), young adult (*n* = 15), and aged (*n* = 21) (Brown–Forsythe and Welch ANOVA, age group effect, *p* = .0067; multiple *t*-tests, young vs. aged, *p* = .0418). Estimation plot (right) for current area of ACh responses in young vs. aged adult type II hair cells (D). Representative aged adult type II hair cell response to 300 µM ACh (*n* = 3) in control media, 100 nM apamin (SK channel blocker), and 100 nM apamin +1 µM strychnine (α9/10 nicotinic receptor antagonist) held at −44 mV (top traces), −64 mV (middle traces), and −94 mV (lower traces) (E). ACh = acetylcholine, ANOVA = analysis of variance.

ACh-evoked currents in type II HCs were significantly reduced in aged compared to young adult mice (Figure 5B–D). This included both negative peak current amplitude at −64 mV (*I*_ACh_; Figure 5C), and the net charge at −64 mV (Figure 5D; aged *n* = 21 vs young adult *n* = 15; *p* < .05). At a membrane potential of −44 mV, the outward current was abolished by SK channel antagonist, apamin (100 nM; Figure 5E, top traces). The remaining inward current was blocked by addition of strychnine, a potent blocker of α9-containing nicotinic receptors (Figure 5E, purple trace). Similarly, at the near-resting membrane potential of −64 mV, apamin blocked the outward current, revealing a large inward current that was subsequently blocked by strychnine (Figure 5E, middle traces). At a membrane potential of −94 mV, apamin partially blocked the inward current, whereas the remaining inward current was blocked by strychnine (Figure 5E, lower traces).

## Discussion

Aging in humans is associated with general vestibular decline paralleled by an increased risk of falls (32). Mechanisms of vestibular decline are not well understood but the peripheral sensory organs of the inner ear that detect and electrochemically encode head motion are implicated (33). Although balance decline has previously been linked to neuroanatomical changes, such as HC loss and reductions in the density of afferent neurons, a consensus on the impacts of aging on HCs has been difficult to reach. For example, in earlier human studies, HC loss was not evident until the sixth and eighth decades in the cristae and otoliths, respectively (34). More recent work suggested progressive loss of vestibular HCs from birth, as opposed to later loss (35). In contrast with these 2 studies, a stereological study of cristae found minimal HC loss until the 10th decade (2). Differences in sample preparation, sample size, and counting methods likely explain these discordant results. Aging-related impacts on peripheral vestibular function are similarly conflicted, with some measures indicating decline as early as the fifth decade (36), whereas others suggest no (10), progressive (37), or late functional decline (38,39). Clearly, more work is needed to resolve the issue of age-related reductions in HC numbers, afferent and efferent pathway connectivity, and how that relates to peripheral vestibular function.

Here, we used an aging colony of C57BL/6 mice that lacks the variables that often confound studies of vestibular function in aging humans, such as genetic variability, ototoxin exposure, inner ear infection damage, and Ménière’s disease. Although C57BL/6 mice exhibit age-related hearing loss due to a *Cdh23* mutation, age-related vestibular decline occurs at a much slower rate and is likely not affected by the mutation (40). Consistent with this, baseline VOR has been shown to be minimally affected in aged C57BL/6 mice from the same colony as used in the present study (12).

Aged mice exhibited significantly more difficulty in navigating a narrow balance beam in comparison to young adult mice. Aged mice performed as well as young adult mice on a wider beam, indicating motor and visual functions are intact. Impairments observed were isolated to the narrow beam, which places greater demands on balance function (Figure 1B). While this behavioral test does not assess peripheral vestibular function in isolation, these results are consistent with a level of balance hypofunction for which the peripheral vestibular system plays an important role. Gait analysis in aged mice during both forced and natural balance behaviors could expand upon these findings in future studies.

**Figure 6.**
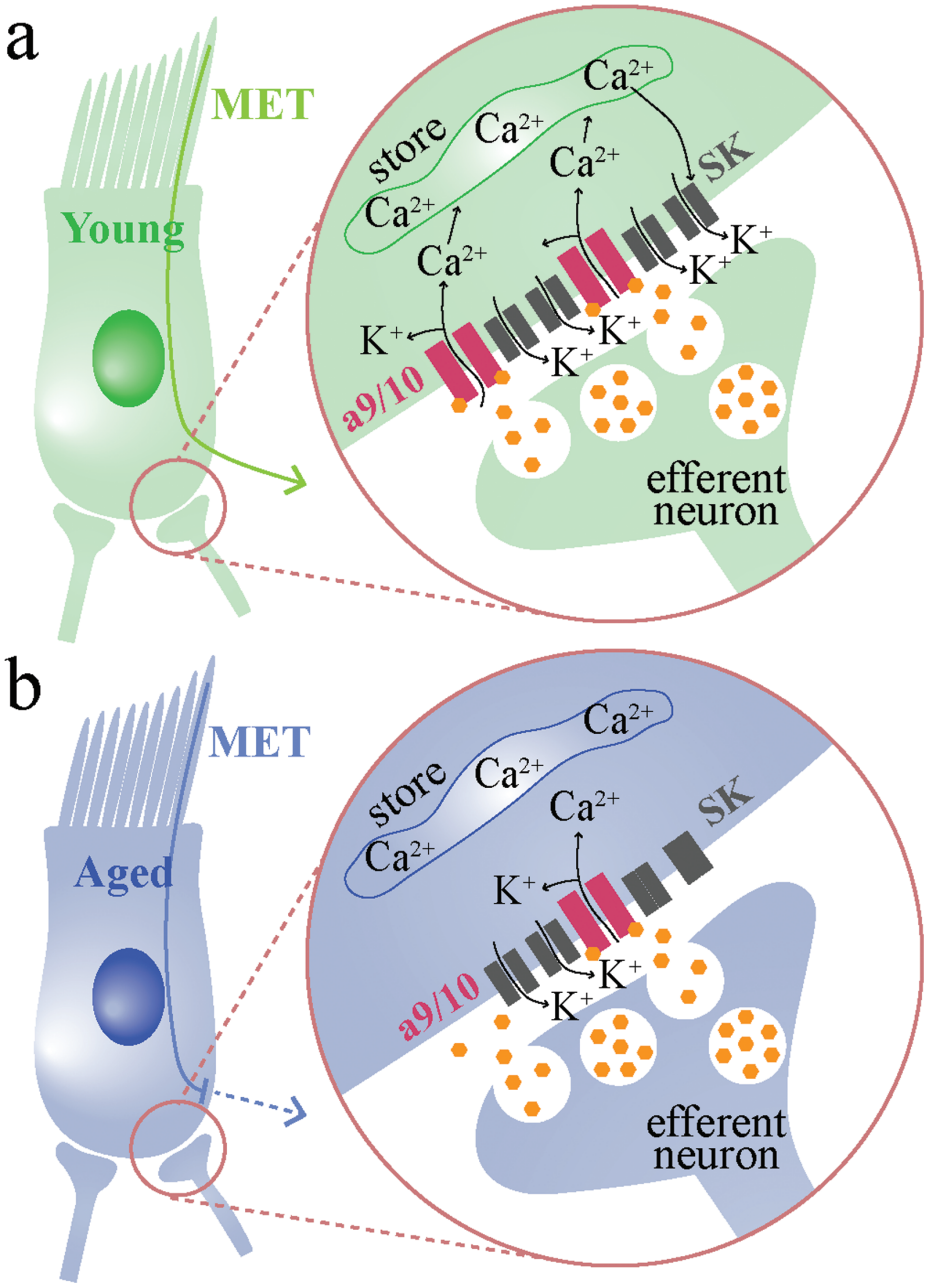
Hypothesis for altered nicotinic modulation of aging type II vestibular hair cells. In young adult type II hair cells (A), mechanoelectrical transduction (MET) currents can be shunted when coincided with large basolateral conductances evoked by ACh. These large basolateral conductances rely upon α9/10 nicotinic ACh receptors (nAChRs), small conductance Ca^2+^-sensitive K+ (SK) channels, and may be amplified via Ca^2+^-induced Ca^2+^ release from subsynaptic cisterns. Lowering of membrane impedance allows for a shift in hair cell gain in response to MET currents. This would impact CaV channel activation, and glutamate release onto primary afferents, thereby modulating head motion signal encoding. In the aged condition (B), where α9/10 nAChR conductance is reduced, there may not be sufficient Ca^2+^ influx to trigger Ca^2+^ release from stores. This would reduce SK current magnitude and duration and would not trigger the same drastic reduction in membrane impedance seen in young type II hair cells. Consequently, hair cell gain in response to MET currents would be less impacted by ACh. This alters the capacity for efferent modulation of hair cell gain (sensitivity). ACh = acetylcholine.

Our molecular assays indicate that *Chrna1*, *Chrna9*, and *Chrna10* expression changes in the aging peripheral vestibular system. Nicotinic ACh receptor subunits α9 (*Chrna9*) and α10 (*Chrna10*) are expressed by vestibular HCs (21,41–43). Given the functional association between α9 and α10 subunits (21), it is not surprising that *Chrna9* and *Chrna10* followed similar expression trends across the 3 age groups examined (Figure 2C). *Chrna9* expression is required for fast cholinergic transmission in type II vestibular HCs, as demonstrated in transgenic knockout mice (18,19). The significant reduction in both *Chrna9* and *Chrna10* expression with aging (Figure 2C) is functionally reflected in whole-cell recordings of type II HCs where ACh exposure failed to elicit robust responses in older mice compared to their younger counterparts (Figure 5). Reduced density of α9/10 subunit-containing nicotinic receptors at efferent synapses would result in decreased Ca^2+^ influx into type II HCs following ACh exposure (Figure 6). Due to the lack of commercially available antibodies for the α9 and α10 subunits, we were unable to confirm receptor loss at the protein level. The analogous pharmacology of aged versus young type II vestibular HCs (Figure 5E) was important to confirm, as altered expression of *Chrna9* and *Chrna10* may have coincided with upregulation of other nicotinic receptor subunits. Despite reduced amplitude of ACh responses (Figure 5C), subsequent blockade by apamin and strychnine (Figure 5E) confirmed that aged murine type II HCs still employ the same “2-channel” mechanism for cholinergic modulation of their activity (18).

Influx of Ca^2+^ through the α9/10 subunit-containing nicotinic receptors, potentially amplified by Ca^2+^ from internal stores, is thought to activate SK2 channels (*Kcnn2*) (18). Another group has indicated a role for BK channels (*Kcnma1*; *Kcnmb4*) in the ACh responses of HCs (44). Given the demonstrated functional relationship between ACh exposure and BK channel conductance, we also measured gene expression levels of *Kcnma1* and *Kcnmb4*, in addition to *Kccn2*. There was no significant change in mRNA expression for the small conductance channel *Kcnn2*, but *Kcnma1* expression was reduced in aged cristae (Figure 2A). This is consistent with an immunohistochemistry study that implies BK channel labeling intensity declines in adult rats (45). The functional consequences of the observed age-related decrease in BK channel expression (*Kcnma1*) were not examined in this study, as brief (100 ms) applications of ACh do not trigger BK currents in murine type II HCs (18).

*Chrna1* expression was significantly decreased in aged neuroepithelia, though expression was stable between juvenile and young adult age groups (Figure 2C). An ionotropic role for α1-containing nicotinic receptors has not been reported in vestibular HCs, so its function could not be reliably tested using patch clamp electrophysiology. Knockout of *Chrna1* does not lead to changes in the formation of efferent synapses in the cochlea, nor does it affect the onset of cholinergic transmission in cochlear HCs of the mouse (46), implying *Chrna1* is not essential for efferent synapse formation. However, reduced *Chrna1* expression may relate to dynamic postsynaptic changes occurring in advanced age and may be important for HC function and viability.

Nicotinic α4 subunits (*Chrna4*) are expressed by vestibular afferent cell bodies in Scarpa’s ganglion, and a4-containing nicotinic ACh receptors are associated with calyx-bearing afferents (47), but not with type I or type II HCs (48–50). Type II HCs, in contrast, express nicotinic α9 and α10 subunits (21,50). Since *Chrna4* expression appears to be unaffected by aging in the crista—despite a parallel decline in *Chrna9* and *Chrna10* expression—fast cholinergic signaling could be differentially impacted by aging in primary afferents (mediated by α4β2 receptors) and HCs (mediated by α9/10 receptors), that is, that cholinergic gene expression changes with aging are HC autonomous. The expression of *Chrna9*, *Chrna10*, and *Chrna4* in type I and type II HCs and primary afferents and the relative changes in expression within each of these components present an interesting avenue for future study.

The lack of age-related differences in *I*_H_ currents, peak *I*_Kv_ amplitude, and tail currents (Figure 4) in type II HCs, but significant declines in mRNA expression of cholinergic receptor subunits and evoked ACh responses support the notion of altered postsynaptic cholinergic signaling, as opposed to a general degradation of HC health and/or nonbiological changes (ie, different recording conditions), or a general reduction in conductances across many channel types. While we measured *I*_KIR_ currents in juvenile, young adult, and aged type II HCs we cannot relate this directly to the reported *Kcnj3* and *Kcnj6* expression (GIRK1 and GIRK2) because these are specialized G-protein activated inward rectifier K^+^ channels. Therefore, hyperpolarization alone does not reliably test functional changes to these particular channels, if expressed in type II HCs. There is very little known about the distribution of GIRK1 and GIRK2 ion channels in the vestibular organs. According to a RNAseq database, *Kcnj3* and *Kcnj6* are minimally expressed in developing, FACS-sorted mouse utricular and cochlear HCs (51). *Kcnj3* and *Kcnj6* were detected in the present study using qPCR which is more sensitive than RNAseq. The youngest age group assayed in this study was the juvenile group with a mean age of P24.8, and the oldest age group included in the SHIELD FACS-sorted RNAseq database is P7 (51). Therefore, although *Kcnj3* and *Kcnj6* were not detected at P7, this does not rule out expression by P21. Despite the lack of changes across the 3 age groups, relative gene expression values for *Kcnj3* and *Kcnj6*, together with *Hcn1* and *Hcn2*, are shown in Figure 2B to emphasize the age-related changes to cholinergic postsynaptic signaling genes whereas expression of other ion channel genes remains stable.

We found no gross qualitative changes to presynaptic efferent anatomy in the crista with age (Figure 3). This is consistent with the reported findings of no significant loss of vestibular efferent cell bodies in the aged gerbil brainstem (26). Our anatomical data also support an EM study that reported no changes to synaptic structures in the vestibular organs of 29-month-old C57BL/6 mice (52). In a study that used a BAC transgenic GFP approach to examine *Chrna9* expression in the inner ear, it was shown that efferent terminal structure was not affected by dynamic changes to *Chrna9* expression (43). We have previously shown that homozygous knockout of the α9nAChR subunit did not grossly impact efferent terminal morphology in the crista (18), despite functional changes (22). The lack of overt changes in presynaptic morphology does not rule out alterations in presynaptic neurotransmitter release or postsynaptic signaling with age. A limitation of the present study was the lack of in situ immunolabeling or Western blot analysis (due to the unavailability of a reliable and commercially available antibody) to quantify nicotinic α9 subunit protein levels, which would allow us to better relate mRNA expression changes to in vitro physiological data. Nevertheless, our data point to dynamic changes to cholinergic signaling in mechanosensory HCs that warrant further investigation, in particular given the early synaptopathy in the aging cochlea, including for cholinergic efferents (53–55).

Why vestibular efferent cholinergic signaling should decline with age is not known. It is well established that neurotrophic factor (NTF) signaling is essential for development of inner ear vestibular organs (56). When brain-derived neurotrophic factor (BDNF) is conditionally knocked out in the inner ear, a relatively long-term (6 months) absence of this NTF was associated with a modest but significant decline in HC number, and a marked drop in vestibular ganglion neurons and afferent innervation to the vestibular organs (8). To further assess the possibility for impaired NTF signaling as a contributor to the aging-related cholinergic transmission decline, we assessed expression of BDNF (*Bdnf*), neurotrophin 3 (*Ntf3*), and their cognate receptors TrkB (*Ntrk2*) and TrkC (*Ntrk3*), respectively, in a previous aging vestibular organs microarray study carried out be our group (57). There were no significant aging-related changes in expression of the NTFs nor their receptors (data not shown). Brainstem cholinergic groups are relatively resilient to the effects of aging, with no significant change in cell number (25,58). However, functional impairments in brain cholinergic cells have been reported, including with NTF signaling. Our findings of anatomically unaltered cholinergic terminals and brainstem cholinergic efferent neurons, and no age-related change in peripheral vestibular NTF-related gene expression, are consistent with a model of reduced cholinergic function without overt neurodegeneration.

Reduced ACh-evoked nicotinic responses in aging type II HCs (Figure 5) impact multiple processes, illustrated in Figure 6. First, in young adult type II HCs, ACh triggers a large basolateral conductance, shown electrophysiologically by a transient drop in membrane impedance (18). This reduction in membrane impedance would shift HC gain in response to mechanoelectrical transduction (MET) currents. This provides the EVS with the ability to tune HC sensitivity. Second, in our aging mouse vestibular epithelial preparation, reduced conductance through α9/10nAChRs, which are particularly permeable to Ca^2+^, will lead to smaller SK channel conductances both directly and indirectly. Ca^2+^ influx via α9/10nAChRs serves to activate nearby SK channels directly, but it is thought that the Ca^2+^-activated K^+^ conductances via SK channels are amplified via Ca^2+^-induced Ca^2+^ release (CICR) from cisterns located near the efferent synapse (Figure 6). Reduced *Chrna9* and *Chrna10* mRNA expression, if related to reduced α9/10nAChR density at the efferent–HC synapse, could result in insufficient Ca^2+^ influx to trigger local CICR (Figure 6). These mechanisms, both efferent modulation of HC sensitivity and Ca^2+^-dependent mechanisms at efferent-type II HC synapses, warrant further investigation.

Taken together, our findings support remodeling of cholinergic postsynaptic signaling in type II vestibular HCs as a potential contributing factor to altered peripheral encoding of head motion signals with age.

## Supplementary Material

glad067_suppl_Supplementary_Table_S1

## References

[1] Agrawal Y, Merfeld DM, Horak FB, et al Aging, vestibular function, and balance: Proceedings of a National Institute on Aging/National Institute on Deafness and Other Communication Disorders Workshop. J Gerontol A Biol Sci Med Sci. 2020;75(12):2471–2480. doi:10.1093/gerona/glaa097 32617555 PMC7662183

[2] Lopez I, Ishiyama G, Tang Y, Tokita J, Baloh RW, Ishiyama A. Regional estimates of hair cells and supporting cells in the human crista ampullaris. J Neurosci Res. 2005;82(3):421–431. doi:10.1002/jnr.20652 16211560

[3] Merchant SN, Velazquez-Villasenor L, Tsuji K, Glynn RJ, Wall C, Rauch SD. Temporal bone studies of the human peripheral vestibular system: normative vestibular hair cell data. Ann Otol Rhinol Laryngol Suppl. 2000;181:3–13. doi:10.1177/00034894001090s502 10821229

[4] Richter E . Quantitative study of human Scarpa’s ganglion and vestibular sensory epithelia. Acta Otolaryngol. 1980;90(3–4):199–208. doi:10.3109/00016488009131716 6258381

[5] Bergstrom B . Morphology of the vestibular nerve. I. Anatomical studies of the vestibular nerve in man. Acta Otolaryngol. 1973;76(2):162–172. doi:10.3109/00016487309121495 4771953

[6] Lopez I, Honrubia V, Baloh RW. Aging and the human vestibular nucleus. J Vestib Res. 1997;7(1):77–85. doi:10.3233/VES-1997-7107 9057161

[7] Kevetter GA, Zimmerman CL, Leonard RB. Hair cell numbers do not decrease in the crista ampullaris of geriatric gerbils. J Neurosci Res. 2005;80(2):279–285. doi:10.1002/jnr.20451 15765526

[8] Elliott KL, Kersigo J, Lee JH, Yamoah EN, Fritzsch B. Sustained loss of BDNF affects peripheral but not central vestibular targets. Front Neurol. 2021;12:768456. doi:10.3389/fneur.2021.768456 34975728 PMC8716794

[9] Johnson Chacko L, Blumer MJF, Pechriggl E, et al Role of BDNF and neurotrophic receptors in human inner ear development. Cell Tissue Res. 2017;370(3):347–363. doi:10.1007/s00441-017-2686-9 28924861

[10] McGarvie LA, MacDougall HG, Halmagyi GM, Burgess AM, Weber KP, Curthoys IS. The video head impulse test (vHIT) of semicircular canal function—age-dependent normative values of VOR gain in healthy subjects. Front Neurol. 2015;6:154. doi:10.3389/fneur.2015.00154 26217301 PMC4495346

[11] Anson ER, Bigelow RT, Carey JP, et al Aging increases compensatory saccade amplitude in the video head impulse test. Front Neurol. 2016;7:113. doi:10.3389/fneur.2016.00113 27486430 PMC4947583

[12] Khan SI, Hubner PP, Brichta AM, Smith DW, Migliaccio AA. Aging reduces the high-frequency and short-term adaptation of the vestibulo-ocular reflex in mice. Neurobiol Aging. 2017;51:122–131. doi:10.1016/j.neurobiolaging.2016.12.007 28063365

[13] Lorincz D, Poppi LA, Holt JC, Drury HR, Lim R, Brichta AM. The long and winding road-vestibular efferent anatomy in mice. Front Neural Circuits. 2021;15:751850. doi:10.3389/fncir.2021.751850 35153679 PMC8832101

[14] Simmons, D., J. Duncan, D.C. de Carprona, et al. Development of the inner ear efferent system. In: Ryugo D, Fay R, eds. Auditory and Vestibular Efferents. Springer Handbook of Auditory Research. Springer; 2011.

[15] Mathews MA, Camp AJ, Murray AJ. Reviewing the role of the efferent vestibular system in motor and vestibular circuits. Front Physiol. 2017;8:552. doi:10.3389/fphys.2017.00552 28824449 PMC5539236

[16] Poppi LA, Holt JC, Lim R, Brichta AM. A review of efferent cholinergic synaptic transmission in the vestibular periphery and its functional implications. J Neurophysiol. 2020;123(2):608–629. doi:10.1152/jn.00053.2019 31800345 PMC7132328

[17] Parks XX, Contini D, Jordan PM, Holt JC. Confirming a role for alpha9nAChRs and SK potassium channels in type II hair cells of the turtle posterior crista. Front Cell Neurosci. 2017;11:356. doi:10.3389/fncel.2017.00356 29200999 PMC5696599

[18] Poppi LA, Tabatabaee H, Drury HR, et al ACh-induced hyperpolarization and decreased resistance in mammalian type II vestibular hair cells. J Neurophysiol. 2018;119(1):312–325. doi:10.1152/jn.00030.2017 28978760 PMC6048467

[19] Yu Z, McIntosh JM, Sadeghi SG, Glowatzki E. Efferent synaptic transmission at the vestibular type II hair cell synapse. J Neurophysiol. 2020;124(2):360–374. doi:10.1152/jn.00143.2020 32609559 PMC7500374

[20] Schneider GT, Lee C, Sinha AK, Jordan PM, Holt JC. The mammalian efferent vestibular system utilizes cholinergic mechanisms to excite primary vestibular afferents. Sci Rep. 2021;11(1):1231. doi:10.1038/s41598-020-80367-1 33441862 PMC7806594

[21] Elgoyhen AB, Vetter DE, Katz E, Rothlin CV, Heinemann SF, Boulter J. Alpha10: a determinant of nicotinic cholinergic receptor function in mammalian vestibular and cochlear mechanosensory hair cells. Proc Natl Acad Sci USA. 2001;98(6):3501–3506. doi:10.1073/pnas.051622798 11248107 PMC30682

[22] Hübner PP, Khan SI, Migliaccio AA. The mammalian efferent vestibular system plays a crucial role in the high-frequency response and short-term adaptation of the vestibuloocular reflex. J Neurophysiol. 2015;114(6):3154–3165. doi:10.1152/jn.00307.2015 26424577 PMC4686299

[23] Aramakis VB, Hsieh CY, Leslie FM, Metherate R. A critical period for nicotine-induced disruption of synaptic development in rat auditory cortex. J Neurosci. 2000;20(16):6106–6116. doi:10.1523/JNEUROSCI.20-16-06106.2000 10934260 PMC6772566

[24] Roerig B, Nelson DA, Katz LC. Fast synaptic signaling by nicotinic acetylcholine and serotonin 5-HT3 receptors in developing visual cortex. J Neurosci. 1997;17(21):8353–8362. doi:10.1523/JNEUROSCI.17-21-08353.1997 9334409 PMC6573745

[25] Schliebs R, Arendt T. The cholinergic system in aging and neuronal degeneration. Behav Brain Res. 2011;221(2):555–563. doi:10.1016/j.bbr.2010.11.058 21145918

[26] Radtke-Schuller S, Seeler S, Grothe B. Restricted loss of olivocochlear but not vestibular efferent neurons in the senescent gerbil (*Meriones unguiculatus*). Front Aging Neurosci. 2015;7:4. doi:10.3389/fnagi.2015.00004 25762929 PMC4327622

[27] Luong TN, Carlisle HJ, Southwell A, et al Assessment of motor balance and coordination in mice using the balance beam. J Vis Exp. 2011(49). doi:10.3791/2376 PMC3197288 21445033

[28] Lim R, Kindig AE, Donne SW, Callister RJ, Brichta AM. Potassium accumulation between type I hair cells and calyx terminals in mouse crista. Exp Brain Res. 2011;210(3–4):607–621. doi:10.1007/s00221-011-2592-4 21350807

[29] Vandesompele J, De Preter K, Pattyn F, et al Accurate normalization of real-time quantitative RT-PCR data by geometric averaging of multiple internal control genes. Genome Biol. 2002;3(7):RESEARCH0034. doi:10.1186/gb-2002-3-7-research0034 12184808 PMC126239

[30] Schmittgen TD, Livak KJ. Analyzing real-time PCR data by the comparative C(T) method. Nat Protoc. 2008;3(6):1101–1108. doi:10.1038/nprot.2008.73 18546601

[31] Pyott SJ, Meredith AL, Fodor AA, Vázquez AE, Yamoah EN, Aldrich RW. Cochlear function in mice lacking the BK channel alpha, beta1, or beta4 subunits. J Biol Chem. 2007;282(5):3312–3324. doi:10.1074/jbc.M608726200 17135251

[32] Agrawal Y, Carey JP, Della Santina CC, Schubert MC, Minor LB. Disorders of balance and vestibular function in US adults: data from the National Health and Nutrition Examination Survey, 2001–2004. Arch Intern Med. 2009;169(10):938–944. doi:10.1001/archinternmed.2009.66 19468085

[33] Jacobson GP, McCaslin DL, Grantham SL, Piker EG. Significant vestibular system impairment is common in a cohort of elderly patients referred for assessment of falls risk. J Am Acad Audiol. 2008;19(10):799–807. doi:10.3766/jaaa.19.10.7 19358459

[34] Rosenhall U . Degenerative patterns in the aging human vestibular neuro-epithelia. Acta Otolaryngol. 1973;76(2):208–220. doi:10.3109/00016487309121501 4543916

[35] Rauch SD . Vestibular histopathology of the human temporal bone. What can we learn? Ann N Y Acad Sci. 2001;942:25–33. doi:10.1111/j.1749-6632.2001.tb03732.x 11710467

[36] Agrawal Y, Zuniga MG, Davalos-Bichara M, et al Decline in semicircular canal and otolith function with age. Otol Neurotol. 2012;33(5):832–839. doi:10.1097/MAO.0b013e3182545061 22699991 PMC3376350

[37] Mossman B, Mossman S, Purdie G, Schneider E. Age dependent normal horizontal VOR gain of head impulse test as measured with video-oculography. J Otolaryngol Head Neck Surg. 2015;44:29. doi:10.1186/s40463-015-0081-7 26141721 PMC4506627

[38] Li C, Layman AJ, Geary R, et al Epidemiology of vestibulo-ocular reflex function: data from the Baltimore Longitudinal Study of Aging. Otol Neurotol. 2015;36(2):267–272. doi:10.1097/MAO.0000000000000610 25275869 PMC4297246

[39] Matino-Soler E, Esteller-More E, Martin-Sanchez JC, Martinez-Sanchez J-M, Perez-Fernandez N. Normative data on angular vestibulo-ocular responses in the yaw axis measured using the video head impulse test. Otol Neurotol. 2015;36(3):466–471. doi:10.1097/MAO.0000000000000661 25473958

[40] Mock BE, Vijayakumar S, Pierce J, Jones TA, Jones SM. Differential effects of Cdh23(753A) on auditory and vestibular functional aging in C57BL/6J mice. Neurobiol Aging. 2016;43:13–22. doi:10.1016/j.neurobiolaging.2016.03.013 27255811 PMC4893173

[41] Kong WJ, Cheng HM, Cauwenberge P. van. Expression of nicotinic acetylcholine receptor subunit alpha9 in type II vestibular hair cells of rats. Acta Pharmacol Sin. 2006;27(11):1509–1514. doi:10.1111/j.1745-7254.2006.00423.x 17049129

[42] Lustig LR, Hiel H, Fuchs PA. Vestibular hair cells of the chick express the nicotinic acetylcholine receptor subunit alpha 9. J Vestib Res. 1999;9(5):359–367. 10544374

[43] Zuo J, Treadaway J, Buckner TW, Fritzsch B. Visualization of alpha9 acetylcholine receptor expression in hair cells of transgenic mice containing a modified bacterial artificial chromosome. Proc Natl Acad Sci USA. 1999;96(24):14100–14105. doi:10.1073/pnas.96.24.14100 10570205 PMC24197

[44] Kong WJ, Guo CK, Zhang S, Hao J, Wang Y-J, Li Z-W. The properties of ACh-induced BK currents in guinea pig type II vestibular hair cells. Hear Res. 2005;209(1–2):1–9. doi:10.1016/j.heares.2005.06.001 16005587

[45] Schweizer FE, Savin D, Luu C, Sultemeier DR, Hoffman LF. Distribution of high-conductance calcium-activated potassium channels in rat vestibular epithelia. J Comp Neurol. 2009;517(2):134–145. doi:10.1002/cne.22148 19731297 PMC3033495

[46] Roux I, Wu JS, McIntosh JM, Glowatzki E. Assessment of the expression and role of the alpha1-nAChR subunit in efferent cholinergic function during the development of the mammalian cochlea. J Neurophysiol. 2016;116(2):479–492. doi:10.1152/jn.01038.2015 27098031 PMC4978794

[47] Holt JC, Kewin K, Jordan PM, et al Pharmacologically distinct nicotinic acetylcholine receptors drive efferent-mediated excitation in calyx-bearing vestibular afferents. J Neurosci. 2015;35(8):3625–3643. doi:10.1523/JNEUROSCI.3388-14.2015 25716861 PMC4339364

[48] Wackym PA, Popper P, Lopez I, Ishiyama A, Micevych PE. Expression of alpha 4 and beta 2 nicotinic acetylcholine receptor subunit mRNA and localization of alpha-bungarotoxin binding proteins in the rat vestibular periphery. Cell Biol Int. 1995;19(4):291–300. doi:10.1006/cbir.1995.1071 7613517

[49] Zoli M, Le Novere N, Hill JA, Jr., Changeux JP. Developmental regulation of nicotinic ACh receptor subunit mRNAs in the rat central and peripheral nervous systems. J Neurosci. 1995;15(3 Pt 1):1912–1939. doi:10.1523/JNEUROSCI.15-03-01912.1995 7891142 PMC6578133

[50] Hiel H, Elgoyhen AB, Drescher DG, Morley BJ. Expression of nicotinic acetylcholine receptor mRNA in the adult rat peripheral vestibular system. Brain Res. 1996;738(2):347–352. doi:10.1016/s0006-8993(96)01046-3 8955534

[51] Scheffer DI, Shen J, Corey DP, Chen Z-Y. Gene expression by mouse inner ear hair cells during development. J Neurosci. 2015;35(16):6366–6380. doi:10.1523/JNEUROSCI.5126-14.2015 25904789 PMC4405555

[52] Park JC, Hubel SB, Woods AD. Morphometric analysis and fine structure of the vestibular epithelium of aged C57BL/6NNia mice. Hear Res. 1987;28(1):87–96. doi:10.1016/0378-5955(87)90156-0 3038820

[53] Sergeyenko Y, Lall K, Liberman MC, Kujawa SG. Age-related cochlear synaptopathy: an early-onset contributor to auditory functional decline. J Neurosci. 2013;33(34):13686–13694. doi:10.1523/JNEUROSCI.1783-13.2013 23966690 PMC3755715

[54] Liberman LD, Liberman MC. Cochlear efferent innervation is sparse in humans and decreases with age. J Neurosci. 2019;39(48):9560–9569. doi:10.1523/JNEUROSCI.3004-18.2019 31628179 PMC6880465

[55] Grierson KE, Hickman TT, Liberman MC. Dopaminergic and cholinergic innervation in the mouse cochlea after noise-induced or age-related synaptopathy. Hear Res. 2022;422:108533. doi:10.1016/j.heares.2022.108533 35671600 PMC11195664

[56] Fritzsch B, Silos-Santiago I, Bianchi LM, Fariñas I. The role of neurotrophic factors in regulating the development of inner ear innervation. Trends Neurosci. 1997;20(4):159–164. doi:10.1016/s0166-2236(96)01007-7 9106356

[57] Bigland MJ, Brichta AM, Smith DW. Effects of ageing on the mitochondrial genome in rat vestibular organs. Curr Aging Sci. 2018;11(2):108–117. doi:10.2174/1874609811666180830143358 30777575 PMC6388513

[58] Pereira PA, Coelho J, Silva A, Madeira MD. Effects of aging on the cholinergic innervation of the rat ventral tegmental area: a stereological study. Exp Gerontol. 2021;148:111298. doi:10.1016/j.exger.2021.111298 33652122

