# Supplementary material for: Molecular and Functional Changes to Postsynaptic Cholinergic Signaling in the Vestibular Sensory Organs of Aging C57BL/6 Mice
Source: J Gerontol A Biol Sci Med Sci. 2023 Feb 25;78(6):920–9. doi: 10.1093/gerona/glad067 (PMC10235202; doi:10.1093/gerona/glad067)
Supplement: glad067_suppl_Supplementary_Table_S1 [file glad067_suppl_supplementary_table_s1.pdf]

Table S1 – Aliases, primer sequences, and average cycle threshold (Ct) values for genes assayed by qPCR in the mouse crista ampullaris. Ct values reported below have not been normalized to the geometric mean of the housekeeping genes (28s, Actb, and Gapdh). Juvenile mice (n = 8) were 3 weeks of age, young adult mice (n = 8) were 3 months of age, and aged mice (n = 8) were > 24 months of age.

Gene information		Primer sequences		Age group (Ct ± SEM)		
**Symbol**	**Description**	**Forward**	**Reverse**	**Juvenile**	**Young adult**	**Aged**
Kcnma1	Large conductance Ca^{2+}-activated K^+ channel (BK)	GGGCCAGTCTGTCTCATTCC	CTGCTTGGCCCATTCTAGTCA	32.7 ± 0.9	32.0 ± 0.3	32.6 ± 0.4
Kcnmb4	BK channel regulatory beta subunit	GTGCTCCTATATCCCGCCCT	GACCACGATGAGAACACCCA	31.3 ± 0.6	31.3 ± 0.4	31.6 ± 0.4
Kcnn2	Small conductance Ca^{2+}-activated K^+ channel 2 (SK2)	TATCTTCGGCATGTTCGGCA	CTGTATTTCCCTGGCGTGGT	27.2 ± 0.2	26.7 ± 0.1	26.5 ± 0.1
Hcn1	Hyperpolarization activated cyclic nucleotide gated channel 1	TATGAGCACCGATACCAAGGC	CGGCGTTAGCAAAAAGAGGC	29.2 ± 0.6	28.1 ± 0.3	28.4 ± 0.4
Hcn2	Hyperpolarization activated cyclic nucleotide gated channel 2	CCACATGACCTACGACCTGG	AAGAGCGCGAACGAGTAGAG	32.6 ± 0.5	31.7 ± 0.4	31.8 ± 0.4
Kcnj3	Inwardly rectifying potassium channel subfamily J member 3 (Kir3.1, GIRK-1)	GCAAGCTGCTCAAATCTCGG	CATGCTTCGCTGGGATAGGT	32.1 ± 0.2	31.3 ± 0.3	30.7 ± 0.3
Kcnj6	Inwardly rectifying potassium channel subfamily J member 6 (Kir3.2, GIRK-2)	GGTACTACACAGGGGACGAC	CCCCACAAGATCTCACTGGT	29.4 ± 0.2	28.5 ± 0.1	28.5 ± 0.1
Chrna1	nicotinic acetylcholine receptor alpha 1 subunit	CATCGAGGGCGTGAAGTACA	CAATGAGCCGACCTGCAAAC	30.4 ± 0.6	29.8 ± 0.5	30.3 ± 0.4
Chrna4	nicotinic acetylcholine receptor alpha 4 subunit	GATGTGGTCCTTGTCCGCTT	GCGGATGGAGGTGACATTCT	30.4 ± 0.5	29.0 ± 0.3	29.0 ± 0.3
Chrna9	nicotinic acetylcholine receptor alpha 9 subunit	CAGTATGACGGGCTGGACTC	CATCCACCACACAGGAGCTT	29.8 ± 0.3	28.7 ± 0.1	29.2 ± 0.1
Chrna10	nicotinic acetylcholine receptor alpha 10 subunit	GGCCCTAATGCACATCCAGT	GCTGGGGGCTGACTCTAATG	27.1 ± 0.5	26.8 ± 0.9	26.3 ± 0.1
